# More than a cool illusion? Functional significance of self-motion illusion (circular vection) for perspective switches

**DOI:** 10.3389/fpsyg.2015.01174

**Published:** 2015-08-10

**Authors:** Bernhard E. Riecke, Daniel Feuereissen, John J. Rieser, Timothy P. McNamara

**Affiliations:** ^1^Space Lab, School of Interactive Arts and Technology, Simon Fraser University, Surrey CampusSurrey, Canada; ^2^Cognitive Science, Simon Fraser University, Burnaby CampusBurnaby, Canada; ^3^Psychological Sciences, Vanderbilt UniversityNashville, TN, USA; ^4^Department of Psychology, Psychological Sciences, Vanderbilt UniversityNashville, TN, USA

**Keywords:** spatial updating, self-motion illusion, vection, virtual reality, perspective taking, functional significance of vection, auditory vection, biomechanical vection

## Abstract

Self-motion can facilitate perspective switches and “automatic spatial updating” and help reduce disorientation in applications like virtual reality (VR). However, providing physical motion through moving-base motion simulators or free-space walking areas comes with high cost and technical complexity. This study provides first evidence that merely experiencing an embodied illusion of self-motion (“circular vection”) can provide similar behavioral benefits as actual self-motion: Blindfolded participants were asked to imagine facing new perspectives in a well-learned room, and point to previously learned objects. Merely imagining perspective switches while stationary yielded worst performance. When perceiving illusory self-rotation to the novel perspective, however, performance improved significantly and yielded performance similar to actual rotation. Circular vection was induced by combining rotating sound fields (“auditory vection”) and biomechanical vection from stepping along a carrousel-like rotating floor platter. In sum, illusory self-motion indeed facilitated perspective switches and thus spatial orientation, similar to actual self-motion, thus providing first compelling evidence of the functional significance and behavioral relevance of vection. This could ultimately enable us to complement the prevailing introspective vection measures with behavioral indicators, and guide the design for more affordable yet effective VR simulators that intelligently employ multi-modal self-motion illusions to reduce the need for costly physical observer motion.

## Introduction

When we move through our surroundings, self-to-object relations constantly change in a rather non-trivial manner. In order to not get lost easily, it is essential for moving organisms to remain oriented during locomotion, for example by continuously updating self-to-object relations and thus anticipating perspective switches. For real-world locomotion like walking (even with eyes closed), our ability to do just that is often attributed to a largely automated “spatial updating” of our mental egocentric representation of our immediate surroundings (Rieser, 1989; Presson and Montello, 1994). This updating process ensures that our mental representation stays aligned with our dynamically changing position and orientation in space – even in the absence of direct perceptual support, for example when closing our eyes for a moment or in darkness. When navigating through virtual worlds and computer games, however, we tend to get lost more easily, especially when reliable landmarks are missing. This reduced spatial updating performance in virtual reality (VR) is often attributed to missing biomechanical and vestibular cues accompanying the visually conveyed locomotion (Wraga et al., 2004).

### Can Vection Facilitate Perspective Switches?

One common paradigm for quantifying the ease or difficulty of such spatial updating is to instruct users to adopt a novel perspective conveyed by a VR simulation or verbal instructions (e.g., “imagine facing the door, point to the window”), and subsequently asking them to point to previously learned objects using their hand or a pointing device like a joystick. When perspective switches are only imagined or visually simulated, pointing to previously learned objects tends to be relatively slow, error-prone, and require considerable cognitive effort (Rieser, 1989; Presson and Montello, 1994; Farrell and Robertson, 1998; May, 2004). Conversely, allowing users to physically locomote to the visually simulated or to-be-imagined perspective tends to reduce pointing errors, response times, and perceived cognitive load, even when navigating with eyes closed (Rieser, 1989; Presson and Montello, 1994; Klatzky et al., 1998; Avraamides et al., 2004; Campos et al., 2009; Frissen et al., 2011). That is, perspective switches tend to be facilitated whenever they are supported by physical motion cues supporting an automatic spatial updating of our egocentric mental representation. Here, we investigated if “vection,” that is, the illusory sensation self-motion in the absence of actual self-motion, might be able to provide at least some of the benefits of actual self-motion, but without the need for physical motion (for reviews on vection in the context of VR, see Riecke, 2011; Riecke and Schulte-Pelkum, 2013; Hettinger et al., 2014; Lawson and Riecke, 2014).

If illusory self-motion could indeed facilitate perspective switches, presumably by initiating or supporting (automatic) spatial updating, this might help to reduce the need for physically moving observers in motion simulation applications such as vehicle simulation, architecture walk-throughs, or tele-presence. While common approaches like moving-base motion simulators and free-space walking areas can be quite effective, they come with substantial cost, complexity, and requirements for space and safety measures. Hence, even a slight reduction in the requirements for physical user motion could be of substantial applied benefit. Apart from its applied relevance, showing that vection can facilitate perspective switches would be, to the best of our knowledge, the first clear evidence of the functional or behavioral significance of vection, in the sense that the percept of vection comes with benefitial behavioral consequences, in that it would facilitate perspective switches that are otherwise more difficult to perform^1^. This could also help to bring us closer to devising much-needed, objective behavioral indicators of vection (Palmisano et al., 2015), a phenomenon that is traditionally investigated using introspective measures and is thus potentially prone to experimental demand characteristics or other biases like higher-level/cognitive confounds (Lepecq et al., 1995; Palmisano and Chan, 2004; Riecke, 2009; Riecke and Schulte-Pelkum, 2013; Palmisano et al., 2015). Ultimately, this could also help to shed light on the question posed by Palmisano et al. (2015) whether the conscious sensation of self-motion is just an epiphenomenon and delayed by-product of our brain with little utility or relevance, or can actually affect our behavior.

### Does Vection have any Functional Significance?

In the process of theorizing about potential necessary versus sufficient requirements for differernt types of spatial orientation, von der Heyde and Riecke (2002) and Riecke (2003) proposed that the occurrence of automatic and continuous spatial updating might require the sensation of self-motion, be it mediated by real or illusory self-motion. Here, we asked if the illusory sensation of self-motion might also (at least under some circumstances) be a sufficient prerequisite for automatic spatial updating, in the sense that automatic spatial updating and perspective switches would be facilitated by participants experiencing illusory rotations from their original to the instructed perspective. That is, this study was designed to investigate the potential functional significance or behavioral relevance of vection, a topic that is receiving increasing interest amongst vection researchers, but has to the best of our knowledge never before been convincingly answered (see review in Palmisano et al., 2015). Although Chance et al. (1998) speculated that using a larger-FOV display in their VR spatial updating task might have been sufficient to elicit vection, which in turn could improve path integration and spatial updating performance, vection in their study was not assessed, and their hypothesis was to the best of our knowledge never explicitly tested. Other researchers were more skeptical toward potential benefits of vection: For example, Warren (1995, p. 297) suggested that vection might not have any functional significance: “Complete vection, in which the scene appears stationary and all motion is attributed to the observer, does not occur until 8–12 s after onset. This long time delay casts doubt on the functional significance of the sensation of self-motion in the control of behavior.”

Yet, there is evidence that experimental conditions which are more conducive to vection can also improve performance, whereas conditions where vection is unlikely to occur can systematically increase errors. For example, Grigo and Lappe (1998) showed that heading judgments tend to become less accurate when the optic flow field is presented for shorter durations (0.4 or 0.8 s), which are generally too short to experience any vection. Unfortunately, however, vection in such studies is generally not directly assessed, such that it remains unknown if vection was indeed perceived for the longer durations (1.8 and 3.6 s in the above study) and if it played a causal role. In fact, in most studies that are not directly tailored toward investigating vection, visual simulations are likely insufficient for inducing strong vection, for example because the stimulus presentation duration is too brief, the optic flow is too sparse or intermittent, or the visual field of view (FOV) too small. In such situations, participants can even show drastic and categorical errors such as left–right or up–down reversed heading judgments (Palmisano and Gillam, 2005; Palmisano et al., 2015) and left–right or up–down reversed point-to-origin responses (Riecke, 2008; Gramann et al., 2012; Goeke et al., 2013). Again, vection in these and comparable studies was not directly assessed, such that a potential contribution of vection or the lack of vection remains speculative.

Similarly, reducing the FOV has been shown to impair vection (Brandt et al., 1973; Nakamura, 2008) as well as reduce performance in a variety of behavioral tasks such as locomotion, maneuvering, reaching, or exploring a new environment, leading to increased errors and time required for task completion (Alfano and Michel, 1990; Toet et al., 2007). However, vection is hardly ever assessed in such behavioral tasks. Moreover, correlations do not imply causation, and for tasks like the above-mentioned ones, the manipulated parameter that is expected to enhance vection (e.g., FOV or stimulus duration) very likely also has additional effects that are not mediated by vection, leaving the question open whether vection itself has a causal effect of our behavior and performance.

Visually induced motion sickness also seems to occur more likely in situations where vection is or could potentially be experienced, (Lee et al., 1997; Smart et al., 2002; Riecke, 2011; Keshavarz et al., 2015). While some studies observed positive correlations between the strength and occurrence of vection and visually induced motion sickness (Diels et al., 2007; Palmisano et al., 2007; Bonato et al., 2008), other studies found no such positive correlation (Prothero et al., 1999; Bonato et al., 2009; Keshavarz and Hecht, 2011; Riecke and Jordan, 2015) or correlations that did not reach significance (Keshavarz and Berti, 2014; Keshavarz et al., 2014). Given the current knowledge, it is yet unclear whether vection is indeed causally related to or functionally significant for visually induced motion sickness. For example, while motion sickness tends to occur more frequently when visuo-vestibular cue conflicts are large, vection generally tends to be facilitated when visuo-vestibular cue conflicts are reduced (Kennedy et al., 2003; Palmisano et al., 2007), even though there can be exceptions (Palmisano et al., 2011).

### Methodological Challenges in Providing Evidence for the Functional Significance of Vection

In sum, in order to provide more compelling evidence for a functional significance of vection, one would ideally need to design experiments where the occurrence of vection is the only aspect that is experimentally manipulated in a randomized controlled study. However, the occurrence and strength of vection is largely a result of changes in the sensory stimulation (e.g., stimulus speed or FOV, Brandt et al., 1973; Howard, 1986; Nakamura, 2008) with potential additional contributions of participants behavior (e.g., changes in viewing patterns from smooth pursuit or staring to foreground fixation or free gazing, Fischer and Kornmüller, 1930; Becker et al., 2002; Palmisano and Kim, 2009) or higher-level/top–down contributions (e.g., whether the moving stimulus is interpreted as an natural scene or background motion, or knowing/sensing that actual self-motion is possible, Lepecq et al., 1995; Riecke, 2011; Riecke and Schulte-Pelkum, 2015).

Apart from these factors affecting vection, which in turn may affect behavior, all of these factors could potentially also affect behavior directly or mediated by other mechanisms, such that it is an experimental challenge to manipulate the occurrence and strength of vection while minimizing potential other influences and confounds. For example, increasing the FOV in a motion simulation in VR will likely enhance vection, which might improve spatial orientation performance, but the increased FOV also provides more visual information that could equally benefit spatial orientation directly, without vection meditating the effect.

**FIGURE 1:**
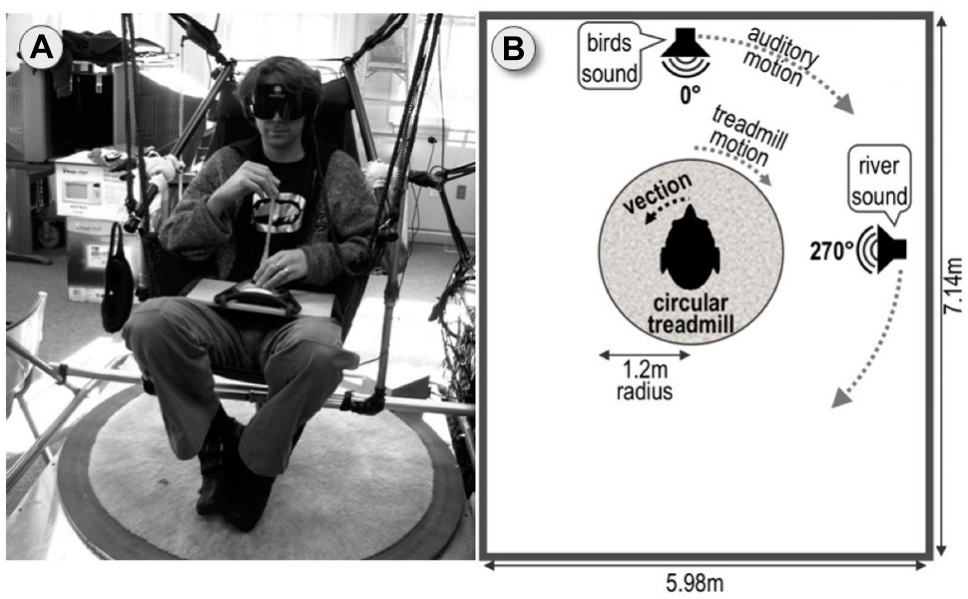
**Experimental setup. (A)** Circular treadmill with hammock chair suspended above. Blindfolded participant wearing noise cancelation headphones points using the joystick. **(B)** Top–down schematic view of the setup.

As an attempt toward addressing this issue, the current study avoided visual vection-inducing cues altogether and instead combined rotating sound fields and biomechanical cues from stepping along a circular treadmill to induce circular vection in blindfolded participants, as illustrated in **Figure 1**. Pre-tests had suggested that unless vection is experienced, these auditory and biomechanical cues by themselves do not provide any benefit for the behavioral task used, namely imagined perspective switches.

**FIGURE 2:**
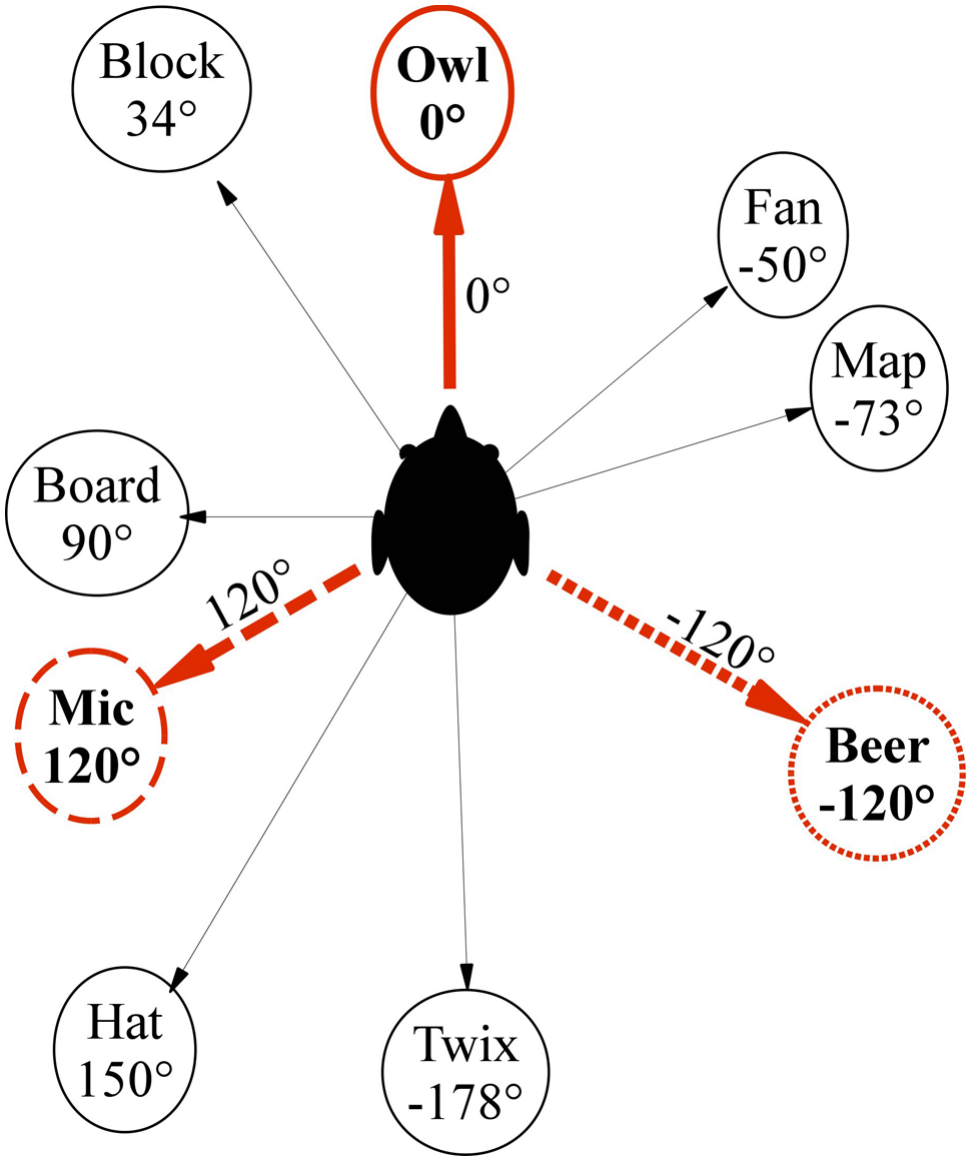
**Top–down schematic view of pointing target layout**.

Before the main experiment, we asked participants in the current study to learn an irregular object layout from one perspective in the lab (cf. **Figure 2**) before being blindfolded and subsequently perform mental perspective switches of 120° and 240° and compared this to the baseline condition of no perspective switch (0°), which was supposed to be easy. To assess if vection can facilitate perspective switches, we compared three motion conditions: Participants were either (a) stationary and asked to imagine the perspective switch (IMAGINE condition), (b) stationary but perceived illusory self-motion (circular vection) to the instructed perspective (VECTION), or (c) were physically rotated to the instructed perspective (REAL ROTATION).

Circular vection is most commonly elicited by moving visual cues. However, similar embodied sensations of self-motion in the absence of actual self-motion can be elicited by non-visual cues as well (Marme-Karelse and Bles, 1977; Bles, 1981), and are increasingly referred to as “vection” even though there is some debate as to how best to define vection (Palmisano et al., 2015). For the purpose of the current study, we combined two non-visual modalities capable of inducing circular vection, audition and biomechanical cues. To this end, blindfolded participants were presented with a combination of sound fields rotating around them (Lackner, 1977; Marme-Karelse and Bles, 1977; Väljamäe, 2009) and biomechanical motion cues from stepping along a rotating floor platter (“circular treadmill,” see **Figure 1**), similar to sitting stationary above a rotating carousel (Bles, 1981). Adding matching rotating sound fields to biomechanical vection induced by a circular treadmill has been previously shown to significantly enhance circular vection (Riecke et al., 2011). Even though visual cues can be very effective in inducing vection (Brandt et al., 1973; Riecke, 2011; Hettinger et al., 2014), we have intentionally excluded visual stimuli from our study because extensive pre-tests have shown that visuals seem to interfere with imagination and perspective-taking tasks (Riecke et al., 2011) and might introduce other confounds.

The study was designed to address three research hypotheses:

#### Hypothesis 1: Imagining a Novel Perspective is Difficult, Even When Supported by Real or Illusory Self-Motion

In order to assess potential facilitation of perspective switches due to vection, we need to create a situation where imagining a new perspective is indeed difficult. This was accomplished by asking participants to learn a fairly large irregular layout of target objects embedded in a natural cluttered lab environment from one learning perspective aligned with the cardinal direction of the rectangular room. Imagining a perspective is generally facilitated when the to-be-imagined orientation matches the learning/experienced orientation or is aligned with the main reference axis used for encoding, an effect called *memory-encoding alignment effect* (Avraamides and Kelly, 2008). Conversely, misalignment is thought to require additional, cognitively effortful transformations or other inference processes before the environment can be imagined and thus represented in working memory in the instructed perspective (Klatzky, 1998). These additional retrieval and transformation/inference costs for misalignment typically lead to increased errors and response times in perspective taking tasks (for reviews, see McNamara, 2003; Avraamides and Kelly, 2008; McNamara et al., 2008). For the current study, this predicts a general performance advantage for the learning perspective of 0° as compared to the non-experienced 120° and 240° perspectives in all motion conditions. Note that we did not predict a performance difference between the 120° and 240° motion conditions, as these are both 120° away from the learning orientation.

#### Hypothesis 2: Vection Facilitates Perspective Switches

Hypothesis 2 addressed the key research question: Can perspective switches be facilitated if supported by the illusory sensation of rotating to the to-be-imagined orientation? That is, we hypothesized improved performance in the VECTION as compared to IMAGINE condition for instructed perspective switches away from the 0° learning orientation. When participants are asked to imagine a perspective switch away from their actual (sensorimotor) perspective, performance typically decreases, and effect that has been attributed to both *mental transformation costs* (Rieser, 1989; Presson and Montello, 1994; Easton and Sholl, 1995) and *sensorimotor interference costs* originating from the conflict between the to-be-imagined versus actual or sensorimotor perspective (Presson and Montello, 1994; May and Wartenberg, 1995; May, 1996, 2000, 2004; Wang, 2005). While the current study was not designed to disambiguate between mental transformation and sensorimotor interference cost, we expected illusory and real rotations in the VECTION and REAL ROTATION conditions to facilitate instructed perspective switches by reducing mental transformation costs (due to eliciting spatial updating that is believed to have low cognitive load) as well as reducing interference costs [as updating one’s mental representations should reduce the conflict between one’s perceived (sensorimotor) and imagined orientation].

#### Hypothesis 3: Illusory Self-Motion is Less Effective in Facilitating Perspective Switches than Actual Self-Motion

There were several reasons why we expected perspective switches (120° and 240° conditions) to be less facilitated in the VECTION as compared to the REAL ROTATION condition. First, illusory self-motion induced by bi-modal (auditory-biomechanical) circular vection does not occur instantaneously with the stimulus onset, but only after a vection onset latency of up to 30 s or more seconds (Bruggeman et al., 2009; Riecke et al., 2011, 2015). Second, auditory-biomechanical circular vection is often not fully saturated and tends to be somewhat less compelling than vection induced by full-field stimulation in an optokinetic drum, where participants sometimes cannot distinguish between illusory and actual self-motion (Brandt et al., 1971, 1973; Palmisano and Gillam, 1998).

## Materials and Methods

### Participants

A total of 17 naive participants (11 female) completed the experiment for standard payment. Four additional participants were excluded, two for not reliably perceiving vection in the pre-screening phase, and two for not following experimental procedures. Participants were between 18 and 47 years old (25.3 years average). All participants had normal or corrected-to-normal vision, normal binaural hearing, and no signs of vestibular dysfunction, as determined by a standard Romberg test (Khasnis and Gokula, 2003). The experiment was IRB-approved and in accordance with the Declaration of Helsinki.

### Stimuli, Task, and Apparatus

#### Circular Treadmill and Setup

Throughout the main experiment, participants wore noise-canceling headphones and blindfolds and were seated on a hammock chair suspended above a motorized, circular treadmill of 1.2 m radius as depicted in **Figure 1A**. A detailed description of the setup can be found in (Riecke et al., 2009a). Although fixed, the hammock chair allowed for slight swaying motions which have been suggested to facilitate vection providing by a cognitive-perceptual framework of movability, (Riecke et al., 2009a; Riecke, 2011).

#### Target Learning and Pointing

The experiment was performed in a cluttered rectangular room of 7.14 m × 5.98 m, in which nine irregularly spaced objects with one-syllable names were selected as pointing target objects (see **Figure 2**). Whereas the majority of prior studies on imagined perspective switches used a small number of regularly arranged target objects in simple and often somewhat artificial environments (Diwadkar and McNamara, 1997; Shelton and McNamara, 1997; McNamara, 2003; McNamara et al., 2008; Marchette and Shelton, 2010), we wanted to test if vection could facilitate perspective switches in an ecologically more valid context, where a larger number of objects is irregularly arranged and embedded into a natural, cluttered room, thus making it less likely that participants could use abstract or higher-level strategies. A learning phase was used prior to the main experiment to ensure that participants could point without vision to all targets within 10° accuracy.

Pointing was performed using a modified wireless Logitech Freedom 2.4 joystick that was positioned on participants’ laps (see **Figure 1A**). To increase ease of pointing and pointing accuracy, the handle of the joystick was replaced by a 200 mm × 9 mm Plexiglas rod. The direction of joystick deflection indicated the pointing direction, and a pointing was recorded once the joystick was deflected by more than 90%. Participants were asked to hold the tip of the joystick handle with their index finger and thumb of their preferred hand using a precision grip (see **Figure 1A**) while holding the basis of the joystick with the other hand.

#### Biomechanical Stimuli

For the VECTION condition, circular biomechanical vection was induced by rotating the circular treadmill while the hammock chair remained stationary and asking participants to step their feet sideways to compensate for the floor’s rotation (Bles, 1981; Riecke et al., 2009a, 2011) (**Figure 2**). Treadmill rotation speed ramped up to 60°/s over 3 s.

For the REAL ROTATION condition, the circular treadmill was kept stationary, and participants were asked to comfortably step along sideways while the experimenter rotated the chair at a speed that matched the average perceived rotation speed in the VECTION condition, which was about 30°/s. That is, participants performed similar sideways walking motions in the VECTION and REAL ROTATION condition.

#### Auditory Stimuli

Auditory vection was induced by participants listening to binaural recordings of what it sounded like to rotate in the actual lab. For generating such vection-inducing auditory stimuli to accompany the biomechanical vection-inducing stimuli, we positioned one speaker directly in front of the observer seated in the hammock chair (0°, 2.3 m away) and a second speaker to their right (270°, 3.3 m away), see **Figure 2**. For the recordings, the 0° speaker displayed a purpose-made mix of 14 bird songs, whereas the 270° speaker displayed a mix of several waterfall and river sounds. These stimuli were chosen in pre-experiments because they could be well localized, easily disambiguated, and were much less disturbing than the white or pink noise stimuli used in many studies. Binaural recordings were collected using miniature microphones (Core Sound Binaural Microphone Set) mounted at the entrance of the ear canal. A more detailed description of the binaural recordings can be found in Riecke et al. (2009a). The binaural recordings of one of the experimenters passively rotating on the circular treadmill with 60°/s while both speakers provided easily localizable sound cues. Note that we did not go through the effort of performing individualized binaural recordings for each participant as a previous study using a similar setup showed that non-individualized binaural recordings were equally effective in inducing auditory circular vection as individualized recordings (Riecke et al., 2009a). For the IMAGINE and REAL ROTATION condition, a non-spatialized (mono) recording of the same sounds was used to mask all sounds from the actual lab without providing any orientation cues.

### Experimental Design

As customary in vection research, a within-participants design was used to reduce issues with the typically large between-subject variability. Each participant completed 32 trials, a factorial combination of three motion conditions (IMAGINE, VECTION, REAL ROTATION) in separate sessions of pseudo-balanced order × three angular disparities angles (0° baseline, 120°, 240°) in randomized order × two turning directions (clockwise/counter-clockwise) × two repetitions per condition (blocked). Turning direction was alternated to balance conditions and to reduce the occurrence of motion sickness and motion after-effects, but was not analyzed separately.

### Procedure

#### Instruction and Target Learning Phase

After signing informed consent, participants were seated on the stationary hammock chair facing the learning orientation of 0° and underwent a training phase to familiarize themselves with the pointing procedure and target layout (see **Figure 2**). Throughout the training phase the hammock chair remained stationary, but participants could turn their head around to see the different targets as needed. After learning the target layout, participants were asked to point to targets announced in random order via headphones until having pointed to each target three times with less than 10° absolute error. Once participants were familiar with the target names and layout, they were asked to close their eyes during target announcement and pointing as participants in the main experiment had to be able to point to targets with eyes closed to ensure that they would be able to point to the targets while blindfolded during the main test. During training, they were free to open their eyes in between trials, though.

#### Main Experiment

Throughout the main experiment participants were blindfolded and wore noise-canceling headphones. To assess if vection would facilitate imagined perspective switches, participants were asked to imagine perspective switches of 120° or 240° away from the learned, default orientation of 0° in three different motion conditions. In an IMAGINE condition, perspective switches had to be performed purely mentally, without any real or illusory self-motion. This was expected to yield the lowest performance. In the VECTION condition, biomechanical and auditory vection-inducing cues were carefully controlled such that participants first perceived one full 360° illusory self-rotation (to make sure that vection was reliable and stable) and then continued to perceive illusory self-rotation until facing the to-be-imagined perspective. Participants perceived orientation was assessed by asking them to use the joystick continuously point toward the 0° object (“owl”). In the REAL ROTATION condition, participants were again exposed to one full 360° rotation before being rotated to the instructed perspective.

#### Perspective Switch Phase

During the VECTION condition, participants were asked to step along with the platform disk which was slowly ramped up to 60°/s over the course of 3 s while headphones displayed the binaural recording of a sound field that rotated with the same velocity. To be able to track participants’ perceived orientation in the lab, they were asked to use the joystick to continuously point toward the 0° object (“owl”) during the illusory self-rotation. Using the joystick responses we could confirm that participants perceived vection in all trials, with vection onset times averaging around 3 s, and values ranging from immediate vection onset to more than 20 s (SD: 3.1 s). In addition, participants verbally indicated when they approached the “owl” and the to-be-imagined object. Just before the end of one full illusory self-rotation (indicated by almost a 360° joystick rotation), the computer announced the to-be-imagined facing target (i.e., “imagine facing owl” for to-be-imagined headings *H*_TBI_ = 0°, or “imagine turning counterclockwise until facing mic” for *H*_TBI_ = 120°), and the experimenter smoothly decelerated the treadmill such that it came to a complete stop when participants’ perceived orientation (as indicate by the joystick) matched the to-be-imagined facing direction (*H*_TBI_ = 0°, 120°, or 240°). The experimenter was extensively trained to be able to manually control the treadmill speed such that participants perceived self-rotation of either 360° (baseline condition), 360° + 120°, or 360° + 240°. The rotating sound field was cross-faded to the non-spatialized (mono) recording as the platform was slowed down to avoid any auditory orientation cues during pointing yet provide a masking sound to cover potential ambient sounds from the lab.

For the REAL ROTATION condition, the platform disk remained stationary while the chair rotation was controlled by the experimenter to yield a velocity profile matching the VECTION condition. Participants were asked to comfortably step along sideways while rotating to provide biomechanical cues. We expect spatial updating to occur in this REAL ROTATION condition and facilitate the pointing task (Rieser, 1989; Presson and Montello, 1994; Klatzky et al., 1998; Avraamides et al., 2004). Note that the biomechanical cues were similar to the ones in the VECTION condition to allow for direct comparisons. As in the VECTION condition, participants were instructed to continuously point toward the 0° object such that the experimenter could estimate their perceived orientation throughout the trial. Again, just before the end of one full self-rotation (indicated by almost a 360° joystick rotation), the to-be-facing target was announced via headphones. The chair continued rotating and smoothly decelerated to stop at the to-be-imagined orientation (*H*_TBI_ = 0°, 120°, or 240°), resulting in total turning angles of 360° + 0°, 360° + 120°, or 360° + 240°.

A similar procedure was used for the IMAGINE condition, but with the chair and platform remaining stationary thus not providing any vection-inducing auditory or biomechanical stimuli. Instead, participants were presented with mono recordings to mask any external sounds and asked to step in place for comparability until asked to point. They were not asked to imagine a 360° rotation before asked to imagine the perspective switch.

#### Pointing Phase

Immediately after the previous perspective switch phase, participants used the joystick to point, in randomly determined order, to six of the nine target objects announced consecutively via headphones. Participants were asked to point “as accurately and quickly as possible, without sacrificing accuracy for speed.” They never received feedback about their pointing performance during the main experiment.

#### Post-Trial Re-Orientation and Feedback Phase

At the end of each trial, participants were asked to remove headphones and blindfold and re-orient in the room. This served to re-anchor them to the default orientation in the lab. To ensure that participants were always physically facing the default 0° orientation when having their eyes open, they were slowly rotated back to the original 0° orientation after each physical rotation trial before removing the blindfold. Participants were then asked to provide two verbal ratings. For **Task difficulty** they were asked “how difficult was it to imagine the new perspective, on a scale from 0 (quite easy) to 100% (quite hard)?” The **Realism/compellingness of rotating in the lab** was assessed by asking them “how compelling or realistic was the sensation of rotating in the actual lab, on a scale from 0 (not compelling/realistic at all) to 100% (fully compelling/realistic)?” After being instructed about the upcoming trial, participants initiated a trial by putting on the blindfold and headphones, pointing toward the default orientation (“owl” object at 0°), and telling the experimenter that they were ready for the upcoming trial.

### Dependent Measures

From the pointing data we derived four different measures intended to quantify different aspects of spatial updating and the difficulty of perspective switches. The **response time** was defined as the time between the beginning of the target pronunciation (which was adjusted to 500 ms for all targets) and the subsequent pointing, and is typically assumed to indicate the ease of access of our mental representation from the to-be-imagined orientation and the potential degree of interference between the actual/perceived and to-be-imagined orientation. The **absolute pointing error** was used to assess how accurately participants knew where they were with respect to specific objects of interest. To quantify the consistency of participants’ spatial knowledge of the target configuration, the **configuration error** was computed as the mean angular deviation (which is the circular statistics analog to the linear SD) of the signed pointing error, taken over the six pointings (Batschelet, 1981). This configuration error is a measure of the inconsistency when pointing to multiple targets and is independent of the overall heading error. **Absolute heading error** was defined as the absolute value of the mean signed pointing error over the six pointings per trial, and was used to estimate participants overall heading error.

## Results

**FIGURE 3:**
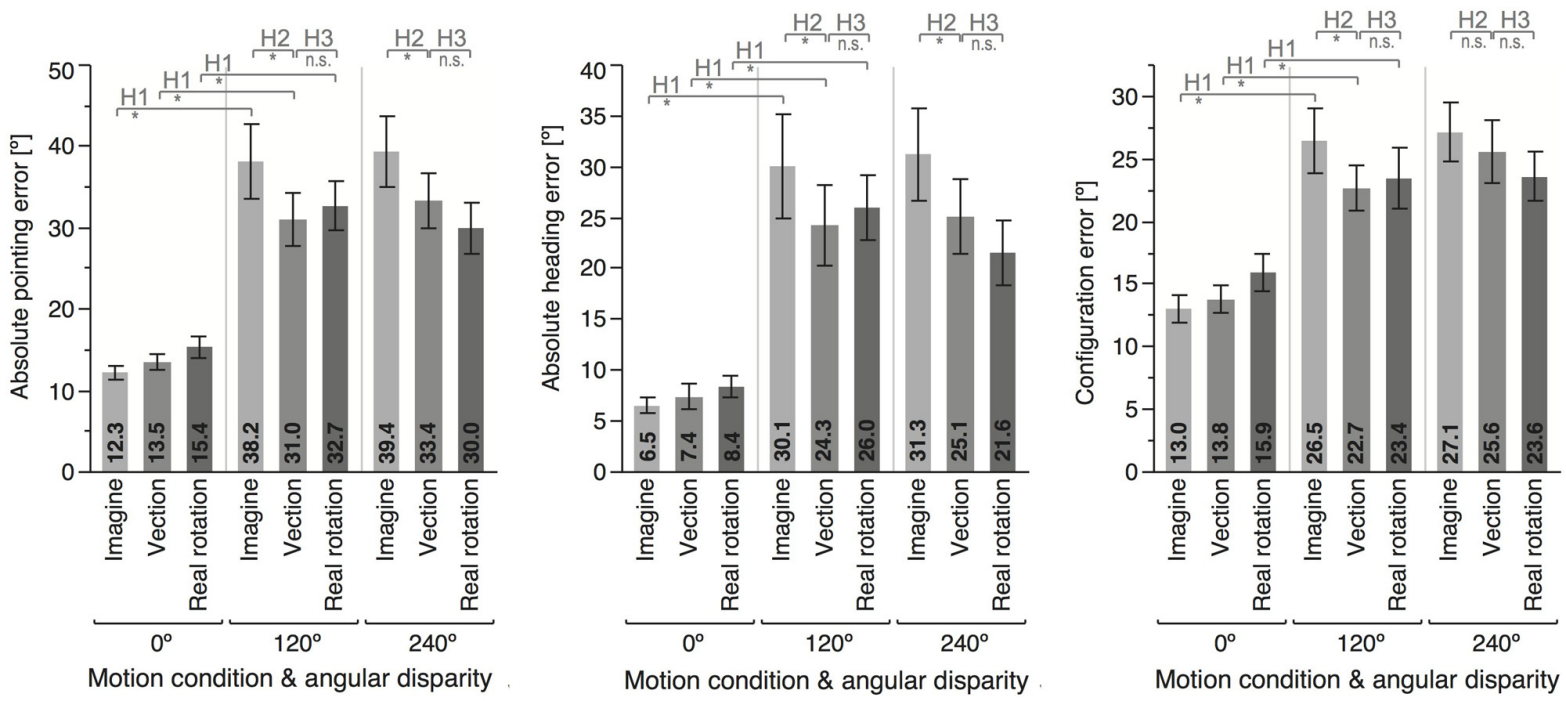
**Means and error bars (±1 SEM) for the different motion conditions and angular disparities**. Top insets indicate whether the planned contrast testing the different hypothesis reached significance (^∗^) or not (n.s.).

**FIGURE 4:**
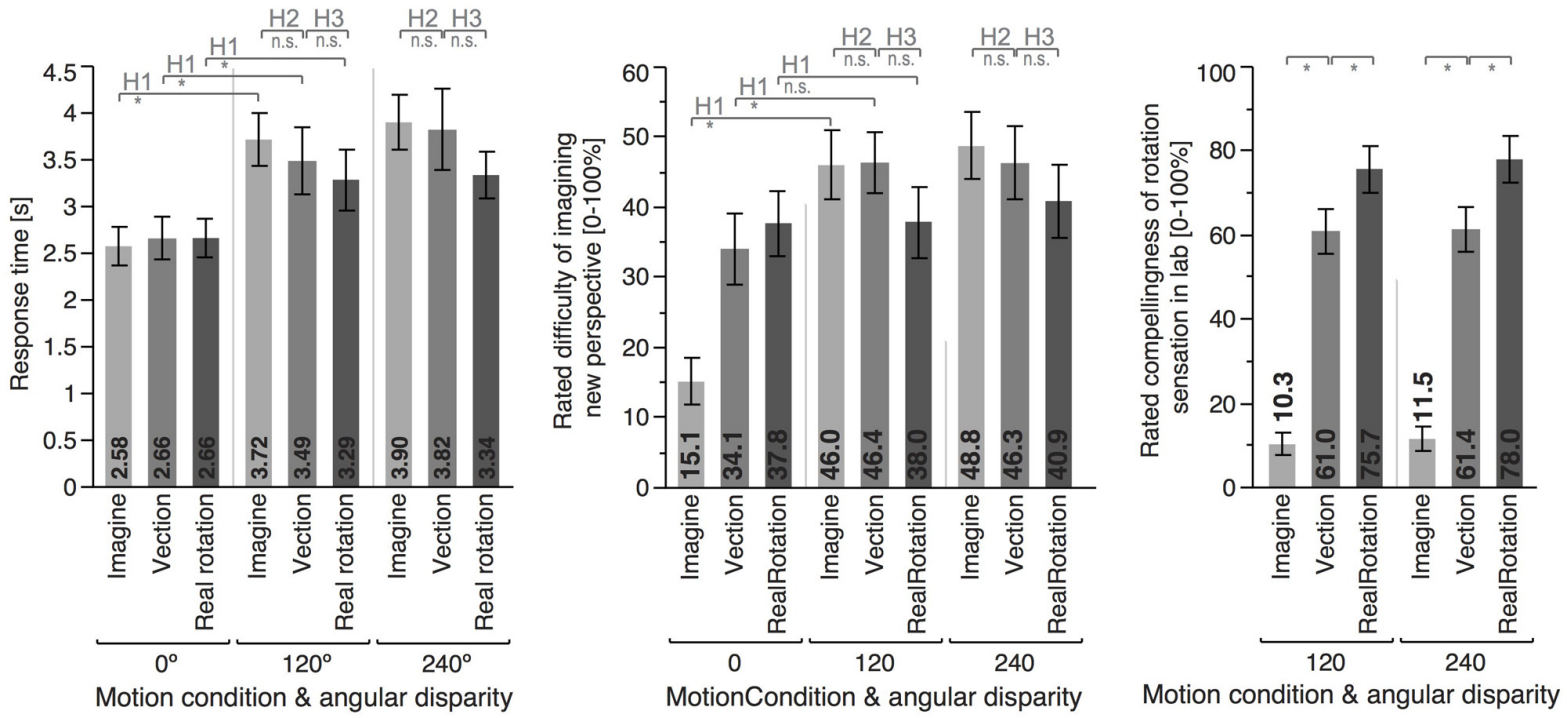
**Means and error bars (±1 SEM) for the different motion conditions and angular disparities**. Top insets indicate whether the planned contrast testing the different hypothesis reached significance (^∗^) or not (n.s.).

Data are summarized in **Figures 3** and **4** and were analyzed using repeated-measures ANOVAs for the independent variables motion condition (IMAGINE, VECTION, and REAL ROTATION) and angular disparity (0°, 120°, and 240°), for each of the dependent variables as summarized in **Table 1**. Planned contrasts were used to test hypotheses 1–3 and are presented in **Tables 2** and **3**.

**Table 1:** ANOVA table of main effects and interaction.

	Main effect: motion condition			Main effect: angular disparity			Interaction		
	*F*(2,32)	*p*	$\eta_{p}^{2}$	*F*(2,32)	*p*	$\eta_{p}^{2}$	*F*(4,64)	*p*	$\eta_{p}^{2}$
Absolute pointing error	**5.151**	**0.001**	**0.244**	**42.641**	**<0.001**	**0.727**	**6.316**	**<0.001**	**0.283**
Configuration error	0.937	0.378	0.055	**37.833**	**<0.001**	**0.703**	**2.869**	**0.030**	**0.152**
Absolute heading error	3.094	0.059	0.162	**25.129**	**<0.001**	**0.611**	**4.710**	**0.009**	**0.227**
Response time	1.547	0.228	0.088	**22.190**	**<0.001**	**0.581**	1.815	0.164	0.102

*Significant effects are boldfaced. Greenhouse–Geisser correction was applied where needed*.

**Table 2:** Planned contrasts for Hypothesis 1.

	H1: IMAGINE, 120° – 0°			H1: VECTION, 120° – 0°			H1: REAL ROTATION, 120° – 0°		
	*F*(1,16)	*p*	$\eta_{p}^{2}$	*F*(1,16)	*p*	$\eta_{p}^{2}$	*F*(1,16)	*p*	$\eta_{p}^{2}$
Absolute pointing error	**30.665**	**<0.001**	**0.657**	**44.968**	**<0.001**	**0.738**	**36.069**	**<0.001**	**0.693**
Configuration error	**29.298**	**<0.001**	**0.647**	**34.223**	**<0.001**	**0.681**	**12.792**	**0.003**	**0.444**
Absolute heading error	**20.452**	**<0.001**	**0.561**	**19.346**	**<0.001**	**0.548**	**27.454**	**<0.001**	**0.632**
Response time	**18.499**	**0.001**	**0.536**	**17.139**	**0.001**	**0.517**	**5.503**	**0.032**	**0.256**

*Significant effects are boldfaced*.

**Table 3:** Planned contrasts for Hypothesis 2 and 3.

	H2: VECTION – IMAGINE, 120°			H2: VECTION – IMAGINE, 240°			H3: REAL ROTATION – VECTION, 120°			H3: REAL ROTATION – VECTION, 240°		
	*F*(1,16)	*P*	$\eta_{p}^{2}$	*F*(1,16)	*p*	$\eta_{p}^{2}$	*F*(1,16)	*P*	$\eta_{p}^{2}$	*F*(1,16)	*p*	$\eta_{p}^{2}$
Absolute pointing error	**14.124**	**0.002**	**0.469**	**5.327**	**0.035**	**0.250**	0.879	0.362	0.052	1.902	0.187	0.106
Configuration error	**4.722**	**0.045**	**0.228**	0.351	0.562	0.021	0.270	0.611	0.017	1.398	0.254	0.080
Absolute heading error	**7.362**	**0.015**	**0.315**	**4.777**	**0.044**	**0.230**	0.842	0.372	0.050	1.414	0.252	0.081
Response time	1.556	0.230	0.089	0.113	0.742	0.007	0.413	0.530	0.025	2.168	0.160	0.119

*Significant effects are boldfaced*.

Angular disparity showed significant effects on all dependent variables (cf. **Table 1**; **Figures 3** and **4**), whereas motion condition showed significant effects only on absolute pointing error. These main effects were qualified by significant interaction for all dependent variables but response time.

### Hypothesis 1: Imagining a Novel Perspective Reduces Performance, Even When Supported by Real or Illusory Self-Motion

As predicted, 120° perspective switches lead to decreased performance compared to the 0° condition for all motion conditions (IMAGINE, VECTION, and REAL ROTATION), indicated by significantly increased absolute pointing errors, configuration errors, and absolute heading errors, as well as reduced response times (cf. **Table 2**; **Figures 3** and **4**). Effects sizes $\eta_{p}^{2}$ were overall large and accounted for 26 – 74% of the variability in the data (Cohen, 1988). Participants rated the 120° condition as more difficult than the 0° condition for both IMAGINE and VECTION conditions (IMAGINE: *F* = 27.444, *p* < 0.001, $\eta_{p}^{2}$ = 0.632; VECTION: *F* = 26.274, *p* < 0.001, $\eta_{p}^{2}$ = 0.622), but not for the REAL ROTATION condition (*F* = 1.695, *p* = 0.211, $\eta_{p}^{2}$ = 0.096).

### Hypothesis 2: Vection Facilitates Perspective Switches

Comparing the 120° perspective switches showed significant facilitation in the VECTION compared to the IMAGINE conditions, indicated by a reduction in absolute pointing error, configuration error, and absolute heading error (see **Figure 3** and **Table 3**). Absolute pointing errors in the IMAGINE condition were 23% higher than in the VECTION condition.

The corresponding effect size $\eta_{p}^{2}$ is 0.469, indicating that experiencing vection during the imagined perspective switch accounted for 46.9% of the variability in the data, which is considered a large effect size (Cohen, 1988). Similarly, absolute heading error was increased by 24% in the IMAGINE condition, with a large effect size of 31.5%, and configuration error was increased by 17%, with a medium effect size of 22.8% (Cohen, 1988). That is, experiencing illusory rotation to the to-be-imagined perspective lead to more consistent and accurate pointing behavior. Response times showed similar trends, but did not reach significance (*p* = 0.230). The 240° condition showed similar trends for vection facilitating perspective switches, which reached significance for the absolute pointing error and absolute heading error, but not for configuration error or response time (see **Figure 3** and **Table 3**). Interestingly, even though 120° and 240° perspective switches were facilitated in the VECTION compared to the IMAGINE condition, task difficulty ratings did not differ significantly and averaged between 46 and 49% (120°: *F* = 0.008, *p* = 0.926, $\eta_{p}^{2}$ = 0.001; 240°: *F* = 0.239, *p* = 0.632, $\eta_{p}^{2}$ = 0.015). As expected, the baseline 0° condition showed no significant differences between the VECTION and IMAGINE condition for any of the dependent measures (all *p*’s > 0.32).

In conclusion, the data supports the hypothesis that vection facilitates perspective switches, in that there was a noticeable performance advantage in the VECTION condition compared to the IMAGINE condition for both 120° and 240° instructed perspective switches, but no differences for the 0° baseline condition. This suggests that imagined perspective switches can (at least under some conditions) indeed be facilitated by illusory self-motion. This confirms Hypothesis 2, and provides the first direct evidence for the functional or behavioral significance of vection.

### Hypothesis 3: Illusory Self-Motion is Less Effective in Facilitating Perspective Switches than Actual Self-Motion

Performance in the VECTION condition did not differ significantly from the REAL ROTATION condition for any of the dependent measures, as detailed in **Table 3**, **Figures 3** and **4**. That is, Hypothesis 3 was not supported, and the perspective switches (120° and 240° conditions) were no less facilitated in the VECTION as compared to the REAL ROTATION condition. Task difficulty ratings averaged between 38 and 46% and showed no significant differences between VECTION and REAL ROTATION for either the 120° perspective switch (*F* = 3.095, *p* = 0.098, $\eta_{p}^{2}$ = 0.162) or the 240° perspective switch (*F* = 0.915, *p* = 0.353, $\eta_{p}^{2}$ = 0.054). Together, this suggests, at least for the task at hand, that illusory self-motion (here: circular vection induced by auditory-biomechanical cues) provided a similar benefit for imagined perspective switches as actual self-motion, and resulted in comparable cognitive load.

### Rating of Rotation Compellingness

Participants rated their sensation of rotating in the actual lab as most compelling or realistic in the REAL ROTATION condition (77.7%), followed by the VECTION condition (61.2%), and least compelling or realistic in the IMAGINE condition (10.9%), see **Figure 4**. Interestingly, even though participants physically rotated in the REAL ROTATION condition, they did not rate this rotation as 100% realistic or compelling, which might be related to the circular treadmill setup and them knowing that the floor could potentially move, even though it never did during REAL ROTATION conditions.

## Conclusion

When navigating through our surroundings, self-to-object relations constantly change and need to be updated so our mental spatial representation stays in alignment with our current position and orientation. For physical locomotion, this is facilitated by an automatic spatial updating process that requires little cognitive load or effort (Rieser, 1989; Presson and Montello, 1994; Farrell and Robertson, 1998). However, when physical motion cues are missing, as is the case in most affordable VR simulations, cognitive load can increase and we tend to get disoriented more easily, which might be attributed to an impaired automatic spatial updating process. Similarly, whereas imagined perspective switches are difficult (especially when they include imagined observer rotations), they become much easier and less error-prone when participants physically move to the to-be-imagined perspective, even with eyes closed (Rieser, 1989; Presson and Montello, 1994; Farrell and Robertson, 1998; Wraga et al., 2004). The current study was designed to test if merely perceiving an embodied illusion of moving to a novel perspective might provide similar facilitation of perspective switches as physical locomotion. That is, we used a perspective-taking task to assess the potential behavioral significance of vection.

Using circular vection induced by biomechanical and auditory cues, our data showed that imagined perspective switches in blindfolded observers were indeed facilitated when participants experienced illusory self-rotation to the instructed perspective (VECTION condition) as compared to merely imagining the perspective switch (IMAGINE condition). Moreover, perspective switch performance in this VECTION condition did not differ significantly from a REAL ROTATION condition where participants were physically rotated to the to-be-imagined perspective.

As discussed earlier, we propose that two factors might have contributed to the observed facilitation of perspective switches in the VECTION condition. First, vection might have reduced ***interference costs*** (May, 2004; Wang, 2005). That is, the conflict or interference between one’s cognitive (to-be-imagined) perspective and the sensorimotor (perceived) perspective might be largely reduced or even disappear when participants experience an embodied (although illusory) rotation to the instructed perspective, as the self-motion illusion presumably rotated their sensorimotor or perceived heading to match the instructed heading. This notion is supported by anecdotal observations of participants being surprised to still face the original orientation in the room after taking took off the blindfold after VECTION trials (Riecke, 2011).

Second, the reason why vection facilitated perspective switches might also be related to vection reducing ***transformation costs*** (Rieser, 1989; Presson and Montello, 1994; May, 2004; Wang, 2005). That is, experiencing illusory self-motion might have facilitated the necessary mental spatial transformation similar to physical motion cues eliciting automatic spatial updating, as was originally proposed by von der Heyde and Riecke (2002) and Riecke (2003).

While it seems likely that experiencing compelling self-motion illusions can reduce both interference and transformation costs, our study was not designed to disambiguate between these two mechanisms, and further research is needed to investigate this. Irrespectively, the finding that vection can, at least to some degree or under some conditions, provide similar behavioral benefits as physical observer motion is promising for a wide range of VR applications ranging from vehicle simulation to architecture walk-throughs, entertainment, tele-operation and tele-presence, where allowing for unrestricted observer motion is costly and often unfeasible. In conclusion, our data suggests that self-motion illusions are not only compelling embodied illusions, but that they can, at least under certain conditions, provide behavioral benefits similar to actual self-motion, thus demonstrating the functional significance of vection (Palmisano et al., 2015).

While these results are promising, there are also several challenges and limitations to the experimental paradigm and study methodology. In order to ensure that the stimuli used to elicit vection did not interfere with the task of imagining a novel perspective, we opted to blindfold people during the experiment and elicited vection using only non-visual cues, namely auditory and biomechanical cues. It is feasible that we would have observed different or more pronounced effects if we had used full-field visual stimuli that are known to be capable of provide compelling self-motion illusions that can sometimes be indistinguishable from actual rotations (Brandt et al., 1973; Dichgans and Brandt, 1978; Riecke, 2011; Hettinger et al., 2014), or combined visual cues with auditory or biomechanical cues, which has also been shown to enhance circular vection (Riecke et al., 2011, 2015). We are currently exploring these options, which might also help to reduce vection onset latencies to below the average of 3 s observed in the current study.

It is also conceivable that the active motor control and proprioceptive cues from the foot stepping during the VECTION condition could have somehow directly benefited perspective switches, irrespective of whether or not vection was elicited. As a step toward addressing this concern, participant in the IMAGINE condition were always asked to step in place in a similar frequency as for the IMAGINE and REAL ROTATION condition. None of the participants reported any compelling sensation of self-motion from this stepping-in-place procedure. Together with the experimental results, this suggests that not the mere act of foot stepping, but the directionality of the foot stepping was essential in facilitating perspective switches, potentially mediated by one’s sensation of self-motion. To explicitly test if perspective switches could be facilitated without any active motor engagement, one could consider including an auditory-only condition where vection is induced solely by a rotating sound field. While we considered this option and performed pilot tests with auditory-only stimuli, it turned out to be difficult to reliably elicit strong vection by purely auditory means. This confirmed prior research which had shown that compared to visual or biomechanical vection, which can be quite compelling, auditory vection tends to be much weaker, occur later, and is only reported by about 20–75% of blindfolded listeners (Lackner, 1977; Riecke et al., 2009b; Väljamäe, 2009). This motivated us to combine auditory with biomechanical vection-inducing cues, which has been shown to yield vection that is stronger than in each of the uni-modal conditions (Riecke et al., 2011).

Another challenge was to manually control the circular treadmill such that participants perceived illusory self-rotation ending at the required heading. The experimenter had extensive practice at this task – nevertheless, participants final perceived heading might have been slightly offset from the instructed perspective. According to participants’ verbal reports in the debriefing, this offset was typically unnoticeable and generally less than 30°. As this offset is much below the instructed perspective switch of 120° and 240°, it seems unlikely that this should have critically affected results. If anything, any offset should have decreased pointing performance for the REAL ROTATION and VECTION condition and thus counteracted any facilitating effects of actual or illusory self-motion.

In conclusion, despite methodological challenges the current study directly supports the proposition that vection is not only one of the most compelling and embodied illusions, but can have functional significance and behavioral relevance. That is, when stationary and blindfolded participants were asked to imagine novel perspectives, they responded more accurately and consistently whenever experiencing illusory self-motion to the to-be-imagined perspective. While further research is needed to corroborate these effect, it suggests that we might not always need to allow for full physical observer motion or costly motion platforms to circumvent the user disorientation and reduced task performance in VR and tele-presence applications – at least for some tasks and scenarios, just providing an embodied illusion of self-motion might suffice. For example, we are currently designing experiments to investigate if categorical errors such as left–right or up–down reversed pointing or heading judgment often observed in VR might be reduced if participants not only see a simulated motion, but also experience self-motion (Riecke, 2008; Gramann et al., 2012; Goeke et al., 2013). Depending on the application scenario, vection could be elicited by any combination of visual, auditory, biomechanical, or vibrational/tactile cues, which have all been found to enhance vection (Riecke, 2011; Riecke and Schulte-Pelkum, 2013; Hettinger et al., 2014; Lawson and Riecke, 2014). While biomechanical cues require some kind of linear, circular, or omnidirectional walking platform or treadmill and can thus be costly to implement, auditory, and visual vection-inducing cues can often be provided at relatively low cost and technical effort, and can be complemented by low-cost vibration elements like shakers or subwoofers. Higher-level factors like providing a cognitive-perceptual framework of movability (i.e., making users believe that actual motion might be possible) or providing naturalistic stimuli of stable landmarks can further enhance vection by affordable means (Lepecq et al., 1995; Palmisano and Chan, 2004; Riecke, 2009; Seno and Fukuda, 2012; Riecke and Schulte-Pelkum, 2013, 2015).

If the functional significance of vection can be replicated in a wider range of experimental paradigms and stimulus conditions, it could enable us to complement the prevailing introspective measures of vection with much-needed behavioral and thus more objective measures of vection (Palmisano et al., 2015). This could ultimately help to devise more reliable measures of vection, as introspective measures are by their nature potentially prone to experimental demand and cognitive and higher-level influences (Lepecq et al., 1995; Palmisano and Chan, 2004; Riecke, 2009; Riecke and Schulte-Pelkum, 2013; Palmisano et al., 2015). Finally, the finding that perspective switches and underlying spatial updating processes were similarly facilitated by real and illusory self-motion are consistent with the proposition that continuous spatial updating might require (and thus imply) the sensation of self-motion, be it mediated by real or illusory self-motion (von der Heyde and Riecke, 2002; Riecke, 2003). In sum, by further studying the functional significance of vection, we hope to not only foster a deeper understanding of underlying processes, but also guide the design of more affordable yet effective VR simulators that intelligently employ multi-modal self-motion illusions to reduce the need for costly physical observer motion.

## Conflict of Interest Statement

The authors declare that the research was conducted in the absence of any commercial or financial relationships that could be construed as a potential conflict of interest.

## Footnotes

1 Part of this paper is based on and extending a short conference paper, © 2012 IEEE, with permission from Riecke et al. (2012). Where methods are identical, some parts have been taken verbatim from the earlier short conference paper.
